# Demographic mechanisms of inbreeding adjustment through extra-pair reproduction

**DOI:** 10.1111/1365-2656.12340

**Published:** 2015-02-24

**Authors:** Jane M Reid, A Bradley Duthie, Matthew E Wolak, Peter Arcese, Martijn van de Pol

**Affiliations:** 1 Institute of Biological and Environmental Sciences, School of Biological Sciences, University of AberdeenZoology Building, Tillydrone Avenue, Aberdeen, AB24 2TZ, Scotland; 2 Department of Forest and Conservation Sciences, University of British Columbia2424 Main Mall, Vancouver, BC, Canada, V6T 1Z4

**Keywords:** inbreeding avoidance, kinship, mate choice, mating system, paternity, pedigree, polyandry, sexual selection

## Abstract

1. One hypothesis explaining extra-pair reproduction is that socially monogamous females mate with extra-pair males to adjust the coefficient of inbreeding (*f*) of extra-pair offspring (EPO) relative to that of within-pair offspring (WPO) they would produce with their socially paired male. Such adjustment of offspring *f* requires non-random extra-pair reproduction with respect to relatedness, which is in turn often assumed to require some mechanism of explicit pre-copulatory or post-copulatory kin discrimination.
2. We propose three demographic processes that could potentially cause mean *f* to differ between individual females’ EPO and WPO given random extra-pair reproduction with available males without necessarily requiring explicit kin discrimination. Specifically, such a difference could arise if social pairings formed non-randomly with respect to relatedness or persisted non-randomly with respect to relatedness, or if the distribution of relatedness between females and their sets of potential mates changed during the period through which social pairings persisted.
3. We used comprehensive pedigree and pairing data from free-living song sparrows (*Melospiza melodia*) to quantify these three processes and hence investigate how individual females could adjust mean offspring *f* through instantaneously random extra-pair reproduction.
4. Female song sparrows tended to form social pairings with unrelated or distantly related males slightly less frequently than expected given random pairing within the defined set of available males. Furthermore, social pairings between more closely related mates tended to be more likely to persist across years than social pairings between less closely related mates. However, these effects were small and the mean relatedness between females and their sets of potential extra-pair males did not change substantially across the years through which social pairings persisted.
5. Our framework and analyses illustrate how demographic and social structuring within populations might allow females to adjust mean *f* of offspring through random extra-pair reproduction without necessarily requiring explicit kin discrimination, implying that adjustment of offspring *f* might be an inevitable consequence of extra-pair reproduction. New theoretical and empirical studies are required to explore the general magnitude of such effects and quantify the degree to which they could facilitate or constrain long-term evolution of extra-pair reproduction.

## Introduction

Identifying the causes of extra-pair reproduction in socially monogamous systems remains a key challenge in behavioural and evolutionary ecology (Jennions & Petrie 2000; Kempenaers 2007; Forstmeier *et al*. 2014). One hypothesis is that individual females mate with extra-pair males to adjust the coefficient of inbreeding (*f*) of extra-pair offspring (EPO) relative to that of the within-pair offspring (WPO) they would produce with their socially paired male (Jennions & Petrie 2000; Foerster *et al*. 2006; Kempenaers 2007; Brouwer *et al*. 2011; Kingma, Hall & Peters 2013; While *et al*. 2014; Reid *et al*. 2015a). By altering offspring *f*, defined as the probability that two homologous alleles present in an offspring will be identical-by-descent, individual polyandrous females could simultaneously alter inbreeding depression in offspring fitness and alter the probability that offspring will inherit alleles identical-by-descent to those carried by the female herself (including alleles influencing reproductive strategy, e.g. Waser, Austad & Keane 1986; Lynch & Walsh 1998; p136; Szulkin *et al*. 2013). Depending on the balance between inbreeding depression and parent–offspring relatedness, either a mean increase or decrease in offspring *f* resulting from extra-pair reproduction could potentially increase or decrease subsequent frequencies of alleles underlying extra-pair reproduction, thereby shaping evolution of reproductive strategy.

Any net change in mean *f* of individual females’ EPO versus their WPO requires some form of non-random extra-pair reproduction with respect to relatedness, such that individual females systematically produce offspring with extra-pair males to whom they are less or more closely related than they are to their socially paired males. This is because completely random and unstructured extra-pair reproduction is not expected to change the *f* of individual females’ EPO versus WPO on average (Appendix S1, Supporting information). The requirement for non-random extra-pair reproduction with respect to relatedness seemingly requires females to assess their relatedness to their socially paired male and potential extra-pair males and allocate offspring paternity accordingly (Wheelwright, Freeman-Gallant & Mauck 2006; Kempenaers 2007; Griffith & Immler 2009). Debates regarding the feasibility of such sophisticated reproductive strategies often focus on individuals’ abilities to discern relatedness via direct auditory, olfactory or molecular cues, thereby enabling pre- or post-copulatory sexual selection for or against related mates (Wheelwright, Freeman-Gallant & Mauck 2006; Kempenaers 2007; Griffith & Immler 2009; Krause *et al*. 2012; Leclaire *et al*. 2013a). It is less commonly considered that aspects of population demography or social structure might create subtle variation in relatedness between subsets of females and males that are available for social pairing versus extra-pair mating, thereby causing the mean difference in *f* between individual females’ EPO and WPO to differ from zero without requiring explicit kin discrimination or direct pre- or post-copulatory selection on relatedness.

Such structure can arise in populations with fine-scale spatial variation in relatedness, where individuals inhabiting proximate breeding locations or groups are more closely related than more distant individuals. Individuals’ relative locations then provide ‘rules of thumb’ by which females could predict relatedness to proximate versus distant extra-pair or extra-group males (Foerster *et al*. 2006; Kempenaers 2007; Szulkin *et al*. 2009; Brouwer, van de Pol & Cockburn 2014; While *et al*. 2014). However, extra-pair reproduction is often highly spatially restricted even within such systems, often involving individuals inhabiting proximate territories (e.g. Wheelwright, Freeman-Gallant & Mauck 2006; Sardell *et al*. 2010; Brouwer *et al*. 2011; Kingma, Hall & Peters 2013; Wang & Lu 2014). The degree to which structured and hence predictable variation in relatedness might also arise among such spatially restricted individuals is rarely considered.

One potential source of structure stems from variation in relatedness among individuals that are available for social pairing versus extra-pair mating at different times within or across reproductive episodes. Many socially monogamous populations where extra-pair reproduction occurs have non-zero adult survival across breeding attempts and years, creating overlapping generations of relatives. Furthermore, social pairings between surviving adults often remain intact across multiple reproductive episodes, creating temporal variation in mate availability (e.g. Jamieson *et al*. 2009; Szulkin & Sheldon 2009; While *et al*. 2014). Here, we first outline three general and non-exclusive processes by which such demography and social structure could cause the relatedness between individual females and their socially paired males to differ from that between these females and their current or future sets of potential extra-pair mates. The mean difference in *f* between individual females’ EPO versus their WPO might then differ from zero given random extra-pair reproduction among temporally constrained sets of potential mates, without necessarily requiring explicit kin discrimination. We then quantify the three processes using long-term pedigree and pairing data from song sparrows (*Melospiza melodia*, Wilson).

### Non-random social pair formation

**Figure 1:**
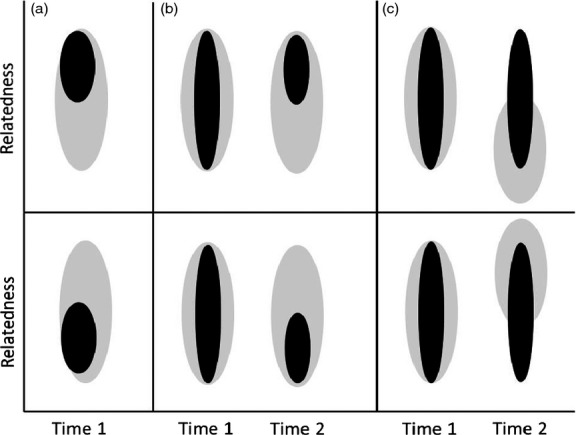
Three processes that could cause a female’s relatedness to a random extra-pair male to differ systematically from her relatedness to her socially paired male, thereby causing a non-zero mean difference in the coefficient of inbreeding (*f*) of extra-pair offspring versus within-pair offspring: a) social pairings (black zone) form non-randomly with respect to the distribution of relatedness among all adults available for pairing (grey zone); b) social pairings (black zone) form randomly with respect to the distribution of relatedness among all adults available for pairing (grey zone, time 1) but then persist non-randomly with respect to relatedness (time 2); and c) social pairings (black zone) form and persist randomly with respect to the distribution of relatedness (grey zone, time 1) but the distribution of relatedness changes for subsequent reproductive attempts (time 2). The three illustrated scenarios are not mutually exclusive and could cause a decrease (upper panels) or an increase (lower panels) in mean offspring *f* given random extra-pair reproduction by socially paired females (black zones) with males drawn from the full distribution of relatedness (grey zones).

First, social pairings might form non-randomly with respect to relatedness, meaning that mean *f* of WPO would differ from that expected given random pairing among all females and males within a particular reproductive episode (Fig.1a, Keller & Arcese 1998; Jamieson *et al*. 2009; Szulkin *et al*. 2009). Random extra-pair reproduction might then alter mean *f* of a female’s EPO compared to WPO produced by non-randomly established social pairings (Fig.1a).

Non-random social pairing could stem from active kin recognition and discrimination, but might also arise passively if there were fine-scale temporal, spatial or social structure in relatedness among adults available for pairing. For example, relatives might tend to reach reproductive condition at similar times, to occupy similar microenvironments or to attain similar social status, potentially due to common genetic, maternal or environmental effects on birth date or ontogeny. Relatives might then be more likely to pair with each other than with unrelated or distantly related individuals with different characteristics (Foerster *et al*. 2006; Reid, Arcese & Keller 2008; Szulkin & Sheldon 2009; Robinson, Kennington & Simmons 2012). Indeed, positive assortative mating with respect to phenotype is widespread in animals (Jiang, Bolnick & Kirkpatrick 2013), causing assortment among relatives given phenotypic resemblance. A female’s relatedness to her socially paired male might then differ systematically from her mean relatedness to the sets of males that become available for extra-pair mating. This is because social pairing typically pre-dates reproduction, particularly in species with biparental territory defence, breeding site construction or nuptial provisioning. Males that were not available for social pairing might therefore become available for extra-pair mating during a female’s fertile period. These males might be less closely related to a female than she is to her socially paired male on average, due to the fine-scale temporal or spatial variation in relatedness that caused assortative social pairing. Random extra-pair reproduction might then cause a directional change in mean offspring *f* (Fig.1a).

### Non-random social pair persistence

Secondly, social pairings might form randomly with respect to relatedness with no active or passive assortative pairing, but the probability that a pairing will persist to subsequent breeding attempts or years might vary with relatedness (Fig.1b). Mean relatedness across social pairings that exist at any time could then deviate from that expected given random pairing at that time, and hence deviate from the mean relatedness between a female and a random extra-pair male. The mean difference in *f* between females’ WPO versus randomly produced EPO could then differ from zero (Fig.1b).

Non-random pair persistence could result from non-random divorce (i.e. separation of paired individuals given that both survive) or non-random adult survival with respect to pair relatedness. Both processes could potentially occur without explicit kin discrimination. Divorce might be more likely following reproductive failure, which might itself result from high social pair relatedness, leading to high offspring *f* and mortality due to inbreeding depression (Kempenaers, Adriaensen & Dhondt 1998; Foerster *et al*. 2006; but see Szulkin & Sheldon 2009; Ihle, Kempenaers & Forstmeier 2013). While an individual adult’s survival might not be expected to be directly affected by its relatedness to its socially paired mate, associations might arise if there were a trade-off between survival and reproductive effort and reproductive effort were to decrease with inbreeding, for example if inbred offspring were more likely to die before the standard termination of parental investment. Furthermore, adult survival and pair relatedness might be correlated if individuals from lineages with high survival probabilities (whether due to genetic or environmental effects) are more likely to pair with a relative, simply because they are likely to have more surviving relatives available for pairing (Reid, Arcese & Keller 2008; Szulkin & Sheldon 2009).

### Temporal variation in relatedness

Thirdly, the difference between a female’s relatedness to her existing socially paired male and her set of potential extra-pair males might change across consecutive reproductive attempts if the frequencies or availabilities of different types of relatives, and the corresponding distribution of relatedness, were to change over time (Fig.1c). For example, when a female first recruits to the breeding population, the males available for social pairing might often include full- and half-brothers and cousins that recruited simultaneously, plus more distant relatives and unrelated individuals. Due to mortality, same-generation relatives will gradually disappear over subsequent reproductive attempts and be replaced by their descendants. If the changing frequencies of these relatives were to cause the mean relatedness between a focal female and her set of potential mates to change, then random extra-pair reproduction could potentially cause a directional change in mean *f* of a female’s EPO versus WPO produced with a previously established socially paired male, even if social pairing was originally random with respect to relatedness (Fig.1c). Similarly, males from previous cohorts might be less likely to be available for social pairing at the time a focal female recruits, because they are more likely to be socially paired already when pairings persist from previous breeding attempts or years. However, they might still be available as potential extra-pair males and might differ in mean relatedness from the set of males available to socially pair with a focal female.

### Empirical test

The hypotheses that random extra-pair reproduction among instantaneously available mates could cause a nonzero mean difference in *f* between individual females’ EPO and WPO given non-random formation (Fig.1a) or persistence (Fig.1b) of related social pairings and/or systematic temporal variation in females’ relatedness to their potential extra-pair mates (Fig.1c) are not mutually exclusive. Even if the overall net difference in offspring *f* was small, it might still be sufficient to influence the evolutionary dynamics of extra-pair reproduction if manifested across numerous generations. To evaluate this possibility, the three processes need to be quantified in populations showing natural variation in demography and relatedness. This can be achieved given data on pairing, mate availability and relatedness without need to assign genetic sires to all offspring.

Long-term pedigree and life-history data from song sparrows inhabiting Mandarte island, Canada, have proved valuable for quantifying mate availability, inbreeding, inbreeding avoidance and inbreeding depression in the wild (Keller 1998; Keller & Arcese 1998; Reid, Arcese & Keller 2006, 2008; Reid *et al*. 2014). Recent analyses showed that female song sparrows were on average slightly more closely related to their socially paired male than to their sets of potential extra-pair males, implying that females would reduce mean offspring *f* through random extra-pair reproduction (mean predicted difference in offspring *f *≈* *0·01, Reid *et al*. 2015a).

Here, we first quantify the distributions of female song sparrows’ coefficients of kinship (*k*) with their sets of potential socially paired and extra-pair males. We then quantify the three processes that could cause mean *f* to decrease between females’ WPO versus EPO given otherwise random extra-pair reproduction among demographically structured sets of potential mates. Specifically, we quantify the degree to which (i) females that formed new social pairings were more closely related to their socially paired male than to other concurrently available males (i.e. non-random pair formation, Fig.1a); (ii) social pairings between closer relatives were more likely to persist to subsequent years (i.e. non-random pair persistence, Fig.1b); and (iii) relatedness changed across years through which social pairings persisted, meaning that females whose social pairings formed in any one year were less closely related to potential extra-pair males available subsequently (Fig.1c).

## Materials and methods

### Study system

Song sparrows are primarily socially monogamous but show occasional social polygyny and polyandry and frequent extra-pair reproduction (Janssen *et al*. 2008; Sardell *et al*. 2010; Hill *et al*. 2011). Both sexes can first breed aged 1 year, have median reproductive life spans of 2 years (maximum 8 years), and pairs can rear up to three broods per year (Smith *et al*. 2006). Socially paired females and males often remain together across breeding attempts within years and across years if both survive (Keller & Arcese 1998). However, both sexes can form new social pairings within or among years following divorce or mortality of their mate (Smith *et al*. 2006).

Since 1975, all breeding territories on Mandarte were monitored, nests were located and all chicks were marked with unique colour-ring combinations *ca*. 6 days after hatching (Smith *et al*. 2006). The occasional immigrants (1·1 year^−1^ on average, sufficient to prevent inbreeding from accumulating) were mist-netted and colour-ringed soon after arriving. All social pairings of adults that bred in each year (i.e. produced at least one clutch) were identified, as were all males that remained socially unpaired when the adult sex-ratio was male-biased (Keller & Arcese 1998; Sardell *et al*. 2010; Reid *et al*. 2014). Adult song sparrows are resident on Mandarte year-round and recruited adults have never been observed to emigrate (Smith *et al*. 2006). The intensive fieldwork ensured an annual resighting probability of *ca*. one. Social pairings that bred are consequently extremely unlikely to have gone unobserved, and the full sets of adult females and males alive in any year are known (Keller & Arcese 1998; Reid, Arcese & Keller 2006).

All song sparrows that survived to adulthood during 1993–2012 were genotyped at 13 highly polymorphic microsatellite loci, and their genetic parents were identified (Sardell *et al*. 2010; Reid *et al*. 2014). Paternities were subsequently verified using up to 170 microsatellite markers and therefore assigned with extremely high confidence. These analyses revealed no extra-pair maternity, but *ca*. 28% of individuals were assigned to extra-pair sires rather than to their mother’s socially paired male and hence identified as EPO (Sardell *et al*. 2010; Reid *et al*. 2014).

A comprehensive pedigree spanning all adult song sparrows alive during 1975–2012 was compiled from all available genetic parentage data and pre-1993 social parentage data (Keller 1998; Reid *et al*. 2014). Standard pedigree algorithms were used to calculate the pairwise coefficient of kinship (*k*) between all adult females and males alive in each year. The coefficient *k* is a measure of relatedness; it quantifies the probability that two homologous alleles sampled from two individuals will be identical-by-descent relative to the defined pedigree baseline, and equals *f* of resulting offspring. Immigrants to Mandarte were assumed to be unrelated to existing natives upon arrival (*k *=* *0, Reid, Arcese & Keller 2006; Reid *et al*. 2014).

Analyses of pairing in relation to *k* were restricted to adults alive during 2007–2012. For these years, all adults’ ancestors back to and including all their great-grandparents (i.e. the great-great-grandparents of potential offspring) were genetically verified or immigrants, and most adults had more distant verified ancestors (Reid *et al*. 2015a). This restriction ensured that *k* was estimated with minimal error or bias due to pedigree error or insufficient depth, providing more precise expectations than are typically available for wild populations (Reid *et al*. 2015a).

All social pairings that first formed during 2007–2012 were identified, and statistics describing the distribution of *k* between socially paired females and males (*k*_SOC_) were computed (mean, median, standard deviation (SD), interquartile range (IQR), range and skew).

### Available males

Quantifying whether and how the distribution of *k*_SOC_ observed across newly formed social pairings differs from that expected given random pairing requires a null model that identifies the sets of males available to pair with each female (Keller & Arcese 1998; Reid, Arcese & Keller 2008; Szulkin *et al*. 2009, 2013; Rioux-Paquette, Festa-Bianchet & Coltman 2010). In practice, the exact sets of available males are hard to define in any wild population. Given social monogamy, the available individuals will depend on the order in which other pairings form and dissolve, which is not readily observable. However, when all population members can be identified, as in Mandarte’s song sparrows, global sets of available individuals can be defined. Any deviation between the observed distribution of *k*_SOC_ and that expected given the global null model can then be investigated, and would potentially suggest some form of truly non-random pairing and/or that there are additional constraints on mate availability that bias the pattern of otherwise random pairing with respect to relatedness (Reid, Arcese & Keller 2008; Szulkin *et al*. 2009).

Global sets of adult male song sparrows that were available for social pairing in a particular year were defined as all individuals that had newly recruited (i.e. age 1 year) and hence were newly available for pairing; plus older individuals that had changed their socially paired mate since the previous year, whether due to divorce or mortality of their previous mate or because they were previously socially unpaired, and hence must have been available for pairing at some point; plus males that remained socially unpaired and hence were presumably still available for social pairing (hereafter together termed the ‘*new-males*’ set of males, Keller & Arcese 1998; Reid, Arcese & Keller 2008). However, being socially paired does not necessarily constrain a male’s availability as an extra-pair male (or may even facilitate it, Sardell *et al*. 2010). Further global sets of males that were potentially available for extra-pair mating were therefore defined as all adult males alive in a particular year (hereafter the ‘*all-males*’ set, Keller & Arcese 1998; Reid, Arcese & Keller 2008). There is no detectable small-scale structure in settlement or hence kinship within Mandarte (Reid *et al*. 2015a).

To quantify among-female variation in *k* with potential mates, statistics describing the distributions of *k* between each female that formed a new social pairing in each year and each male included in the ‘*new-males*’ and ‘*all-males*’ sets for that year were computed.

### Social pair formation

To quantify the degree to which the distribution of *k*_SOC_ across social pairings that formed during 2007–2012 differed from that expected given random pairing with available males (Fig.1a), we first quantified the distribution of the deviation between observed *k*_SOC_ and each female’s mean *k* with the defined ‘*new-males*’ and ‘*all-males*’ sets of males for the year in which each social pairing formed (*k*_MEAN.NEW_ and *k*_MEAN.ALL_, respectively) such that *k*_DEV.NEW_ = *k*_SOC_–*k*_MEAN.NEW_ and *k*_DEV.ALL_ = *k*_SOC_–*k*_MEAN.ALL_.

However, since the distribution of *k* was skewed and individual males cannot simultaneously form socially monogamous pairings with numerous females, subtle deviations between the observed distribution of *k*_SOC_ and null expectation might not be detected simply by comparing observed *k*_SOC_ to global *k*_MEAN.NEW_ or *k*_MEAN.ALL_. We therefore ran simulations to generate null distributions of *k*_SOC_ arising from random social pairing. Each female that formed a social pairing during 2007–2012 was assigned a random socially paired male drawn without replacement from the ‘*new-males*’ set of available males for the focal year, in a random order. For years when the sex-ratio of individuals available for social pairing was female-biased, females that were not assigned a socially paired male from the ‘*new-males*’ set were assigned a random male from the ‘*all-males*’ set of all adult males alive in the focal year without replacement, thereby creating the degree of social polygyny necessary to ensure that all females were socially paired (as observed, Janssen *et al*. 2008). The coefficient *k*_SOC.RAND_ between a female and her randomly assigned male was calculated, and statistics describing the distributions of *k*_SOC_ and *k*_SOC.RAND_ were compared over 10 000 iterations. To assess the sensitivity of conclusions to the assumed set of available males, simulations were repeated with all females’ mates drawn from the ‘*all-males*’ set.

### Social pair persistence

To quantify whether a difference in mean *k*_SOC_ across extant social pairings compared to that expected given random pairing could stem from non-random pair persistence (Fig.1b), we compared the distributions of *k*_SOC_ between social pairings that formed during 2007–2011 that did and did not persist to a second (subsequent) year up to 2012 and used logistic regression to test whether the probability of pair persistence varied with *k*_SOC_.

Failure of a social pairing to persist across years could stem from mortality of either adult, or from divorce. We therefore tested whether the probability that at least one socially paired adult would die, or the probability of divorce given that both adults survived, varied with *k*_SOC_.

Finally, we compared the distributions of *k*_SOC_ between social pairings that first formed during 2007–2010 and persisted to a second year, and then did or did not persist to a third year up to 2012. No pairings persisted to a fourth year during 2007–2012.

### Temporal variation in relatedness

To investigate whether a female’s relatedness to the male population changed across consecutive years, and hence whether mean *f* of EPO produced through random extra-pair reproduction could differ from that of WPO produced by previously formed social pairings (Fig.1c), we identified females whose social pairings persisted to second and third years and used linear mixed models with fixed effects of the year of pair persistence (i.e. first, second or third year) and random female effects to quantify whether *k*_MEAN.ALL_ changed across successive years. This analysis quantifies within-female variation in *k*_MEAN.ALL_ across years through which social pairings persisted. Each individual female’s observed socially paired male was excluded from the sets of potential mates, meaning that any change in *k*_MEAN.ALL_ relates directly to the change in a female’s relatedness to the set of potential extra-pair males.

Analyses were run in R 2.15.2 (R Core Team 2012). Raw means are presented ± 1SD unless otherwise stated. One immigrant female was excluded from analyses because there was no variation in her assumed *k *=* *0 with all males. Conclusions remained similar when non-territorial floaters were excluded from the sets of available males, and when the potential extra-pair mates of each female were restricted to males inhabiting neighbouring territories (because females were no more closely related to neighbours than to non-neighbours, Reid *et al*. 2015a). For reference, *k *=* *0·25, 0·125 and 0·0625 equate to pairings among outbred full-sibs, half-sibs, first-cousins or equivalent relatives, respectively.

## Results

During 2007–2012, the total numbers of adult female and male song sparrows alive on Mandarte varied from 17 to 37 and 13 to 56 respectively, and the sex-ratio varied from 39% to 65% males (Table S1, Supporting information). The numbers of males deemed available for social pairing (i.e. new recruits and individuals that had changed mates since the previous year or remained socially unpaired) varied from 11 to 46 (Table S1, Supporting information). Substantial proportions of males (81–94%) were therefore available for pairing in any particular year, even without social polygyny (Table S1, Supporting information).

A total of 135 new social pairings formed during 2007–2012, involving 85 individual females and 90 individual males and encompassing 125 female-years. Female and male ages at pairing ranged from 1 to 8 and 1 to 7 years, respectively (median 1 year for both sexes) and were not correlated across new pairings (Spearman rank correlation: r_133_ = 0·05, *P* = 0·59).

### Distributions of kinship

**Figure 2:**
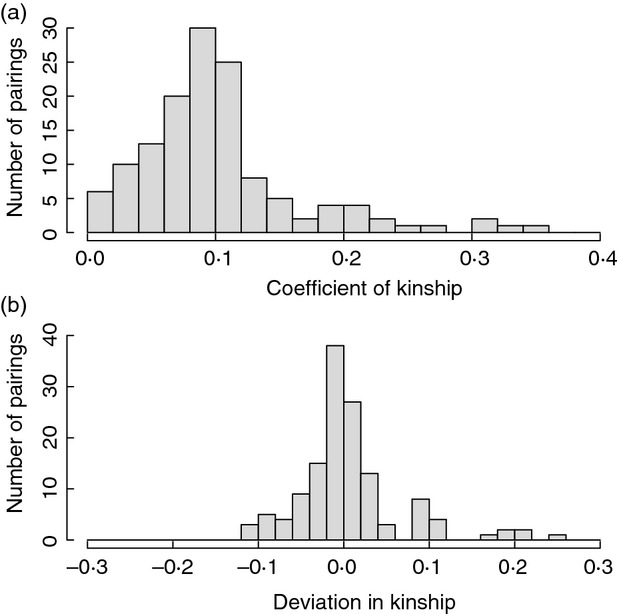
Distributions of a) the coefficient of kinship between a female song sparrow and her observed socially paired male (*k*_SOC_) across 135 social pairings and b) the deviation between *k*_SOC_ and the female’s mean kinship with the ‘*new-males*’ set of males deemed available for social pairing.

Mean *k*_SOC_ across all 135 new social pairings was 0·101 ± 0·064 (median 0·090, IQR 0·065–0·117, range 0·000–0·356, Fig.2a). The distribution of *k*_SOC_ was right-skewed (skew 1·47), and 6 (4·4%) and 23 (17%) pairings were between first-order (*k*_SOC_ ≥ 0·25) and second-order relatives (0·125 ≤  *k*_SOC_ < 0·25), respectively. Mean *k*_SOC_ did not vary significantly across years (linear regression: β_133_ = 0·003 ± 0·003SE, *P* = 0·42) or among years (ANOVA: F_129,5_ = 0·9, *P* = 0·49, Table S1 (Supporting information), conclusions were similar after arc-sin transformation to better approximate normality).

**Figure 3:**
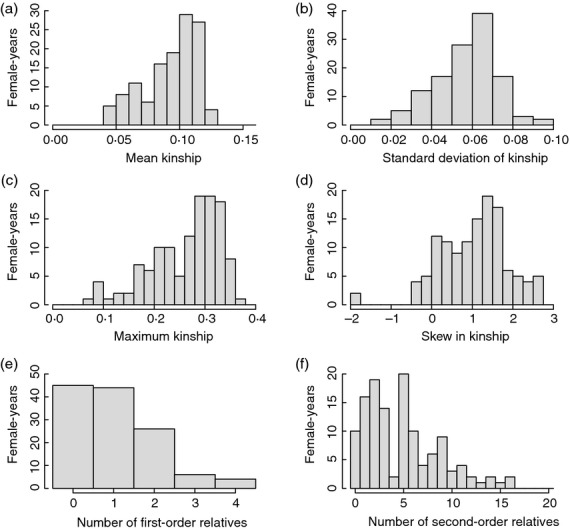
Distributions of the a) mean, b) standard deviation, c) maximum and d) skew in individual female song sparrows’ coefficients of kinship with the ‘*new-males*’ set of males deemed available for social pairing, and the numbers of available e) first-order and f) second-order relatives, across 125 female-years when new social pairings formed.

The distributions of *k* between females that formed new social pairings and the ‘*new-males*’ sets of males deemed available for social pairing in the 125 female-years are summarized in Fig.3 and Table S2 (Supporting information). There was substantial among-female variation in the mean, SD, maximum and skew in *k*, and in the numbers of available first-order and second-order relatives (Fig.3, Table S2, Supporting information). Since minimum *k* was zero in 111 (89%) female-years, most females had some opportunity to pair with an unrelated male (Table S2, Supporting information). Distributions were similar, but not identical, given the ‘*all-males*’ sets of all males alive in each focal year (Fig. S1, Table S2, Supporting information). All females therefore had opportunity for a range of degrees of inbreeding through social pairing and extra-pair reproduction within the defined sets of available males.

### Social pair formation

Across the 135 new social pairings, the deviation between a female’s *k*_SOC_ with her observed socially paired male and her mean *k* with the ‘*new-males*’ set of available males (*k*_MEAN.NEW_) was centred close to zero (mean *k*_DEV.NEW_ 0·007 ± 0·062, median -0·003) but varied among females (IQR −0·021–0·020, range −0·116–0·241) and was slightly right-skewed (skew 1·31, Fig.2b). The distribution was similar when *k*_MEAN_ was calculated across the ‘*all-males*’ set of all males alive in each year (mean *k*_DEV.ALL_ 0·005 ± 0·062, median −0·004, range −0·114–0·235, Fig. S2, Supporting information). Direct comparison between *k*_SOC_ and *k*_MEAN.NEW_ therefore provided no evidence that mean *k*_SOC_ across new social pairings differed from that expected given random pairing.

**Figure 4:**
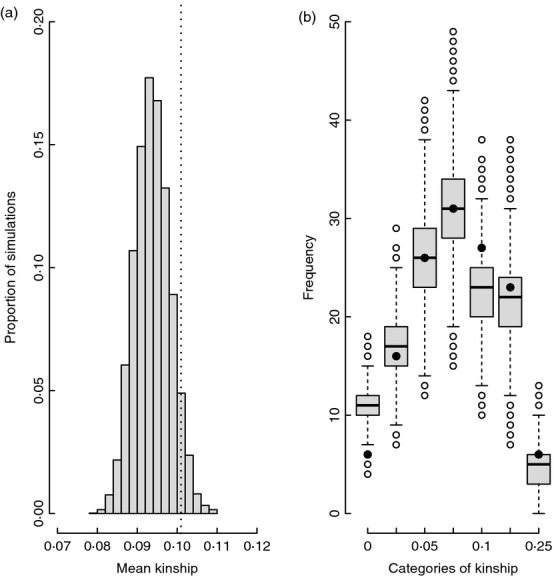
Comparisons between the distributions of a) mean coefficient of kinship (*k*_soc_) between female song sparrows and their observed socially paired males (dotted line) across 135 newly formed social pairings versus a randomly assigned male from the ‘*new-males*’ set of males deemed available for social pairing in each focal year (grey bars) and b) the frequencies of observed (black circles) and simulated pairings falling into categories of *k*_soc_, defined as <0·025, 0·025-0·05, 0·05-0·075, 0·075-0·10, 0·10-0·125 and 0·125-0·25. *X*-axis labels demarcate lower category boundaries. Totals of 6, 16, 26, 31, 27, 23 and 6 pairings were observed within these categories, respectively. Black bars, boxes, whiskers and circles show the median, inter-quartile range, 1·5xIQR and outliers, respectively.

However, simulations that randomly assigned each female a mate from the ‘*new-males*’ set showed that the mean observed *k*_SOC_ of 0·101 tended to be higher than the mean simulated *k*_SOC_ of 0·094 (95%CI 0·085–0·102, *P* = 0·058, Fig.4a). This tendency arose because unrelated and distantly related pairings tended to occur less frequently than expected given random pairing (Fig.4b). Specifically, the observed first quartile value of *k*_SOC_ exceeded the mean simulated value (0·065 versus 0·057, 95%CI 0·050–0·064). The shape of the observed distribution of *k*_SOC_ therefore differed somewhat from that expected given random social pairing (Fig.4b). However, these deviations from expectation were smaller and did not differ from zero when mates were randomly assigned from the ‘*all-males*’ sets of all males alive in each year (mean simulated *k*_SOC_ of 0·096, 95%CI 0·086–0·106, *P* = 0·14, Fig. S3, Supporting information).

### Social pair persistence

Of 103 new social pairings that formed during 2007–2011, 23 (22%) persisted to a second year and 80 (78%) did not. The probability that a pairing would persist did not vary significantly with *k*_SOC_ (Table1a).

**Table 1:** Distributions of the observed coefficient of kinship (*k*_SOC_) across social pairings of song sparrows (a) that formed during 2007–2011 and did and did not persist to a second year, (b) where both adults did or did not survive to a second year, (c) that did and did not divorce given that both adults survived to a second year, (d) that formed during 2007–2010 and persisted to a second year and did and did not persist to a third year and (e) across all social pairings that did or did not persist across consecutive observed years. β ± SE and p are the logistic regression slope and associated standard error and *P*-value. For (d), a t-test rather than logistic regression was implemented due to small sample sizes and non-overlapping distributions of *k*_SOC_, and the t statistic is presented. SD and IQR are the standard deviation and inter-quartile range, respectively

		*N*	Mean ± SD	Median	IQR	Range	β ± SE (p)
a)	Persisted to 2^nd^ year	23	0·110 ± 0·068	0·105	0·061–0·139	0·026–0·308	2·9 ± 3·7 (0·44)
	Did not persist	80	0·098 ± 0·060	0·089	0·065–0·117	0·000–0·301	
b)	Both adults survived	42	0·093 ± 0·067	0·082	0·048–0·117	0·000–0·308	−3·7 ± 3·4 (0·29)
	One or both adults died	61	0·106 ± 0·058	0·097	0·069–0·118	0·000–0·301	
c)	Did not divorce	23	0·110 ± 0·068	0·105	0·061–0·139	0·026–0·308	−9·9 ± 5·8 (0·09)
	Divorced	19	0·073 ± 0·061	0·081	0·033–0·096	0·000–0·254	
d)	Persisted to 3^rd^ year	5	0·159 ± 0·026	0·147	0·142–0·169	0·136–0·200	*t* = −5·9 (0·001)
	Did not persist	11	0·071 ± 0·031	0·067	0·047–0·093	0·031–0·117	
e)	Persisted to next year	28	0·118 ± 0·065	0·115	0·067–0·147	0·026–0·308	6·2 ± 3·4 (0·08)
	Did not persist	91	0·095 ± 0·058	0·086	0·061–0·115	0·000–0·301	

Of the 103 pairings, there were 42 (41%) where both adults survived to a second year and 61 (59%) where one or both adults died. The probability that both adults would survive did not vary significantly with *k*_SOC_ (Table1b).

Of the 42 pairings where both adults survived to a second year, 23 (55%) persisted and 19 (45%) divorced. The probability of divorce tended to decrease with increasing *k*_SOC_, such that mean *k*_SOC_ tended to be higher across pairings that persisted than across pairings that divorced (Table1c).

Of 16 social pairings that formed during 2007–2010 and persisted into a second year, 5 (31%) persisted into a third year and 11 (69%) did not. Mean *k*_SOC_ across pairings that persisted into a third year was higher than across pairings that did not persist after the second year (mean difference 0·088, Table1d). The 11 pairings that did not persist all involved adult mortality rather than divorce. The probability that both socially paired adults would survive from their second year into a third year therefore increased with *k*_SOC_ (Table1d).

Overall, across all 119 observed intervals where social pairings could have persisted to a subsequent year, the probability of persistence tended to increase with *k*_SOC_ such that *k*_SOC_ tended to be higher across pairings that persisted than across pairings that did not (mean difference 0·023, Table1e).

### Temporal variation in relatedness

There were 23 females whose social pairings formed during 2007–2011 and persisted to a second year, and five females whose social pairings formed during 2007–2010 and persisted to a third year. Across these females, mean *k*_MEAN.ALL_ did not differ significantly across the 2 or 3 years through which each female’s social pairing persisted (Table2). Therefore, since *k*_SOC_ is constant within pairings that persisted across years, the difference between *k*_SOC_ and *k*_MEAN.ALL_ could not vary significantly across years. Similarly, the median and IQR of each female’s distribution of *k* with the set of available males did not vary substantially across the years through which a social pairing persisted (Table S3, Supporting information).

**Table 2:** Distributions of the mean coefficient of kinship (*k*_MEAN.ALL_) between female song sparrows whose social pairings persisted to a) a second year (*N* = 23) and b) a third year (*N* = 5) and the set of potential extra-pair males available in the year of pairing and subsequent years. SD and IQR are the standard deviation and inter-quartile range, respectively. *F* values are mixed model test statistics

		Mean ± SD	Median	IQR	Range	
a)	Year of pairing	0·091 ± 0·023	0·102	0·076–0·109	0·047–0·118	*F* = 1·7, *P* = 0·21
	Second year	0·093 ± 0·024	0·100	0·080–0·111	0·043–0·130	
b)	Year of pairing	0·103 ± 0·014	0·108	0·104–0·108	0·080–0·116	*F* = 1·6, *P* = 0·26
	Second year	0·102 ± 0·011	0·101	0·100–0·110	0·086–0·113	
	Third year	0·110 ± 0·009	0·109	0·107–0·110	0·099–0·124	

## Discussion

Extra-pair reproduction is widely hypothesized to allow socially monogamous females to adjust their relatedness to the sire of their offspring and thereby adjust offspring coefficient of inbreeding (*f*, Jennions & Petrie 2000; Kempenaers 2007; Reid *et al*. 2015a). Such adjustments are widely assumed to require explicit pre- or post-copulatory sexual selection for more or less closely related extra-pair males, in turn requiring some mechanism of kin discrimination (e.g. Pizzari, Løvlie & Cornwallis 2004; Griffith & Immler 2009; Jamieson *et al*. 2009; Rioux-Paquette, Festa-Bianchet & Coltman 2010; Brouwer *et al*. 2011; Kingma, Hall & Peters 2013). While individuals of some taxa can distinguish close and/or familiar relatives from non-relatives (Krause *et al*. 2012; Leclaire *et al*. 2013a), it is less clear whether distant relatives might generally be distinguishable from non-relatives and hence whether paternity could be allocated accordingly.

However, the expectation that random extra-pair reproduction will not alter the mean *f* of individual females’ EPO compared to their WPO stems from an assumption that females draw concurrent socially paired and extra-pair males from the same distribution of relatedness (Appendix S1, Supporting information). It is not generally considered that aspects of population demography or social structure might cause systematically diverging sets of related females and males to be available for social pairing versus extra-pair mating at any time, creating diverging distributions of relatedness between individual females and their socially paired versus potential extra-pair males. Random extra-pair reproduction among instantaneously available mates might then cause mean *f* to differ between individual females’ EPO versus WPO, potentially influencing evolution of extra-pair reproduction without necessarily requiring explicit kin discrimination.

Previous analyses showed that individual female song sparrows breeding on Mandarte during 2007–2012 were on average more closely related to their socially paired male than to a random extra-pair male drawn from the concurrent male population, implying that females would reduce mean offspring *f* through random extra-pair reproduction (Reid *et al*. 2015a). Here, we propose and quantify three demographic processes through which such a difference could arise: non-random formation (Fig.1a) or persistence (Fig.1b) of social pairings with respect to coefficient of kinship (*k*), and changing distributions of *k* between females and their potential extra-pair males across years through which social pairings persisted (Fig.1c). We first summarize key results then consider the wider context.

### Social pair formation

Across social pairings that formed during 2007–2012, the distribution of the deviation between a female’s *k* with her socially paired male (*k*_SOC_) and her mean *k* with the ‘*new-males*’ set of potentially available males was centred close to zero. This suggests that mean *k*_SOC_ between social mates, and hence the mean *f* of resulting WPO, did not differ from expectation given random social pairing. This in turn implies an absence of population-wide inbreeding avoidance or preference through social pairing. Keller & Arcese (1998) and Reid *et al*. (2014), Reid, Arcese & Keller (2006, 2008) drew similar conclusions based on earlier years of song sparrow data, albeit estimating *k* from social pedigree data that were not corrected for extra-pair paternity.

However, simulations that sequentially assigned random social mates to individual females from the ‘*new-males*’ set without replacement, thereby mimicking sequential formation of socially monogamous pairings, revealed subtle differences between the observed and expected distributions of *k*_SOC_. Specifically, social pairings among distantly related or unrelated individuals occurred slightly less frequently than expected. This discrepancy between analyses that do and do not account for sequential pair formation (i.e. allocating males without and with replacement) implies that studies that quantify the deviation between observed *k* and the mean calculated across some defined set of available mates (e.g. Jamieson *et al*. 2009; Rioux-Paquette, Festa-Bianchet & Coltman 2010; Billing *et al*. 2012) might not detect subtle patterns of non-random pairing with respect to *k*. The small deviation from expectation could potentially reflect kin discrimination and outbreeding avoidance, but could also result from fine-scale population structure, for example if relatives have similar reproductive timing, social status or other attributes and are therefore more likely to pair with each other than with a non-relative (Reid, Arcese & Keller 2008; Szulkin & Sheldon 2009; Robinson, Kennington & Simmons 2012).

However, the difference between the observed and expected distributions of *k*_SOC_ was smaller when socially paired males were assigned from the ‘*all-males*’ set of all males alive in each focal year rather than the ‘*new-males*’ set of males deemed available for social pairing. Since song sparrows are primarily socially monogamous, males whose existing social pairings persist from previous years might not be fully available to form new social pairings. Assigning social mates from the ‘*all-males*’ set is therefore unlikely to be realistic (Keller & Arcese 1998). However, it illustrates that estimates of the degree of non-random pairing with respect to *k* are somewhat sensitive to assumptions regarding the set of available males (e.g. Szulkin *et al*. 2009). This is in one sense problematic, since the exact set of males available to pair with any individual female will rarely be known in any field system (Jamieson *et al*. 2009), even when all population members are identifiable. Yet it is also interesting because it implies that the distributions of *k* between females and the ‘*new-males*’ versus ‘*all-males*’ sets of males differ to some degree (Table S2, Supporting information). This in turn implies that mean *k* between a female and her socially paired male (assumed drawn from the ‘*new-males*’ set) versus an extra-pair male (assumed drawn from the ‘*all-males*’ set) could potentially differ simply due to population structure rather than necessarily requiring additional forms of non-random extra-pair reproduction.

### Social pair persistence

**Figure 5:**
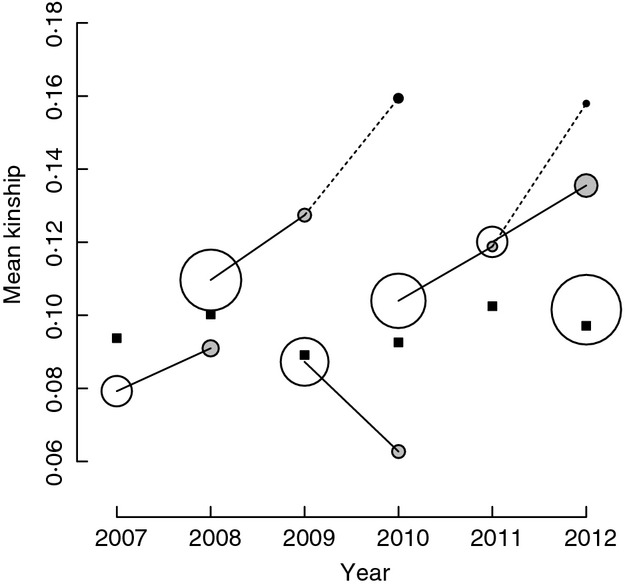
Schematic overview of the mean coefficient of kinship (*k*_SOC_) between a female and her socially paired male across pairings that formed in each year (open circles) and persisted to a second year (grey filled circles) or third year (black filled circles) compared to the grand mean kinship between each female that bred in each year and the set of all available males (i.e. mean *k*_MEAN__.__ALL_, black squares). Solid and dashed lines link pairings that formed in any one year that persisted to second or third years, respectively. Circle sizes represent numbers of pairings. Overall, most observed pairings (circles) fall in the zone where *k*_SOC_ exceeds mean *k*_MEAN__.__ALL_ (above black squares) implying that random extra-pair reproduction would reduce the mean coefficient of inbreeding of females’ offspring.

Across all observed occasions when a song sparrow pairing that formed during 2007–2011 did or did not persist to a subsequent year, social pairings comprising closer relatives tended to be more likely to persist. The overall mean difference in *k*_SOC_ of *ca*. 0·023 between pairings that did and did not persist roughly equates to the difference between pairing with an unrelated individual versus a first-cousin-once-removed (*k *=* *0·031) or a second-cousin (*k *=* *0·016). However, the overall effect did not differ significantly from zero and the two demographic processes underlying pair persistence, namely divorce and mortality, showed opposing patterns. While the probability that both socially paired adults would survive from pairing to a second year did not vary with *k*_SOC_, more closely related socially paired mates that survived tended to be less likely to divorce. Meanwhile, of the social pairings that persisted to a second year, only the more closely related pairings survived to a third year. More years of data are needed to investigate whether such patterns are consistent across years and why they might arise. However the phenomenological conclusion is that, during 2007–2012, the tendency for more closely related social pairings to be more likely to persist to subsequent years contributed to upward drift in mean *k*_SOC_ compared to that observed across newly formed pairings (Fig.5).

### Temporal variation in relatedness

The degree to which an individual adult’s mean relatedness to the opposite sex population changes across years through which previously formed social pairings persist, reflecting changing frequencies of individuals’ ancestors, descendants and other relatives, has not to our knowledge been quantified previously. However, there was no evidence of systematic longitudinal change in a female’s *k*_MEAN.ALL_ with the male population within the duration of individual social pairings. Furthermore, since an adult song sparrow’s annual survival probability is ca. 0·6 (Smith *et al*. 2006), few social pairings persisted for more than 2 years (expected proportion ≈ 0·6^4^(1–d) = 0·13(1–d), where d is the probability of divorce given mutual survival). There was therefore little opportunity for temporal variation in relatedness within extant pairings to substantially influence the mean population-wide difference in *f* between females’ EPO and WPO. In song sparrows, variation in *k*_MEAN_ across years can therefore contribute little to any systematic difference between a female’s *k* with her socially paired versus a random extra-pair male, and hence any systematic difference in offspring *f* that could result from random extra-pair reproduction in post-pairing years.

### Conclusions and context

The previous observation that socially paired female song sparrows would reduce mean offspring *f* through random extra-pair reproduction (Reid *et al*. 2015a) can be qualitatively explained by the combination of slightly non-random social pairing such that pairings among unrelated or distantly related individuals formed slightly less frequently than expected, and a tendency for more closely related social pairings to be more likely to persist to subsequent years (Fig.5). The focus on pairings formed during 2007–2012 ensured sufficient pedigree depth to allow adequately precise estimation of *k* (Reid *et al*. 2015a), but inevitably restricted sample sizes of years and pairings. Further data are therefore required to determine whether observed tendencies for non-random formation and persistence of social pairings with respect to *k* are consistent across years or simply reflect short-term chance events.

Nevertheless, our conceptual framework (Fig.1) and analyses highlight a general need to frame and test hypotheses linking extra-pair reproduction to relatedness in a realistic demographic context. Experimental studies that quantify inbreeding avoidance or preference through multiple mating typically assume that some direct physiological mechanism of kin discrimination exists (e.g. Bretman, Wedell & Tregenza 2004; Pizzari, Løvlie & Cornwallis 2004; Kempenaers 2007; Firman & Simmons 2008). Experimental designs consequently do not incorporate demographic variation or population structure, or hence consider how the distribution of relatedness might vary among temporally varying sets of potential mates. Meanwhile, behavioural ecologists working on wild populations recognize that extra-pair reproduction might depend on aspects of population ecology, including density, habitat geometry and reproductive synchrony (e.g. Kingma, Hall & Peters 2013; Brouwer, van de Pol & Cockburn 2014; Wang & Lu 2014). Furthermore, numerous studies have tested for non-random social pairing or reproduction with respect to relatedness (e.g. Wheelwright, Freeman-Gallant & Mauck 2006; Kempenaers 2007; Jamieson *et al*. 2009; Szulkin *et al*. 2009, 2013; Rioux-Paquette, Festa-Bianchet & Coltman 2010; Billing *et al*. 2012), and some have quantified divorce or widowing rates (e.g. Foerster *et al*. 2006; Kempenaers 2007; Szulkin & Sheldon 2009; Leclaire *et al*. 2013b). However, the potential impact of non-random pair formation and persistence on the degree to which polyandrous females could adjust offspring *f* through instantaneously random extra-pair reproduction is not generally emphasized. Further theoretical and empirical tests of the hypothesis that population demography and social structure might allow females to systematically adjust mean offspring *f* through random extra-pair reproduction among temporally varying sets of potential males are therefore warranted.

Such effects of demography and social structure might be expected to be greatest in populations (i) with fine-scale spatial or temporal variation in reproductive ecology; (ii) where the timing or location of social pairing diverges from that of extra-pair mating; (iii) where adult survival and mate fidelity are sufficiently high for social pairings to persist across multiple reproductive episodes; (iv) where costs of failing to pair or of divorce are high (e.g. given sequential mate searching or requirements for territory acquisition or coordinated parental care); and (v) with overlapping generations, allowing distributions of relatedness to change within the duration of social pairings. Even if net differences in *k* between females and their socially paired versus instantaneously random extra-pair males were small, as in song sparrows, they might potentially facilitate or constrain long-term evolution of extra-pair reproduction if manifested across numerous generations. Indeed, directional adjustment of offspring *f* might be an inevitable side-product of extra-pair reproduction occurring for other reasons and have either positive or negative fitness consequences. Such effects, which could potentially act in conjunction with direct pre- or post-copulatory discrimination among close kin and/or with state-dependent extra-pair reproduction, need to be integrated into hypotheses explaining evolution of extra-pair reproduction in the context of natural population demography, reproductive scheduling and relatedness structure.

## Data accessibility

All data associated with this article are archived at the Dryad Digital Repository: http://dx.doi.org/10.5061/dryad.k8199 (Reid *et al*. 2015b).

## Supporting Information

**Appendix S1.** Expected difference in coefficient of inbreeding of females’ extra-pair offspring versus within-pair offspring given completely random social pairing and extra-pair reproduction. **Fig. S1.** Distributions of coefficients of kinship relative to the ‘*all-male*s’ set. **Fig. S2.** Distribution of the deviation in kinship relative to the ‘*all-male*s’ set. **Fig. S3.** Comparison of observed and simulated kinship relative to the ‘*all-male*s’ set. **Table S1.** Composition of the song sparrow population. **Table S2.** Distributions of coefficients of kinship. **Table S3.** Distributions of coefficients of kinship across years through which social pairings persisted.
