# Supplementary material for: Demographic mechanisms of inbreeding adjustment through extra-pair reproduction
Source: J Anim Ecol. 2015 Feb 24;84(4):1029–40. doi: 10.1111/1365-2656.12340 (PMC4670719; doi:10.1111/1365-2656.12340)
Supplement: Supplementary file 1 [file jane0084-1029-sd1.docx]

**Supporting Information**

**Demographic mechanisms of inbreeding adjustment through extra-pair reproduction**

**Jane M. Reid, A. Bradley Duthie, Matthew E. Wolak & Peter Arcese**

**Appendix S1. Expected difference in coefficient of inbreeding of females’ extra-pair offspring versus within-pair offspring given completely random social pairing and extra-pair reproduction**

Purely random extra-pair reproduction is not expected to change the coefficient of inbreeding (*f*) of females’ extra-pair offspring (EPO) relative to their alternative within-pair offspring (WPO) on average. This is because the distribution of the difference in relatedness between a female and random social versus extra-pair males drawn from the same population, and hence from the same distribution of relatedness, is expected to be centred on zero.

We applied simulations to comprehensive pedigree data from song sparrows (*Melospiza melodia*) to verify the basic expectation that random and unstructured social pairing followed by random and unstructured extra-pair reproduction would not alter mean *f* of females’ EPO versus WPO even given the skewed and irregular distribution of relatedness arising in a natural population (see Fig. 2a, main paper). We thereby verify the expectation that some mechanism of non-random social pairing and/or extra-pair reproduction is required to cause a non-zero difference in mean *f* of EPO versus WPO.

Each female song sparrow that formed a new social pairing during 2007-2012 was assigned a random socially-paired male and a different but otherwise random extra-pair male from the ‘*all males*’ set of all adult males alive in each focal year (i.e. sampling with replacement among females but without replacement within females). The difference in coefficient of kinship (*k*) between each female and her random extra-pair male (*k*_EP.RAND_) versus random socially-paired male (*k*_SOC.RAND_) was calculated as *k*_DIFF.RAND_ = *k*_EP.RAND_ – *k*_SOC.RAND_. Statistics describing the distribution of mean *k*_DIFF.RAND_ across females were summarised over 10000 iterations.

Across the 85 females that formed new social pairings during 2007-2012, the distribution of the difference in *k* between randomly assigned socially-paired and extra-pair males was centred on zero (grand mean of mean *k*_DIFF.RAND_ <0.001 ± 0.004, median <0.001, inter-quartile range -0.002–0.003, range -0.017–0.020, skew < 0.01).

These results confirm the expectation that random social pairing followed by random extra-pair reproduction within the same set of males would not alter the mean *f* of females’ EPO versus WPO, even given the naturally skewed and irregular distribution of *k*.

**Table S1.** **Composition of the song sparrow population.**

For each year during 2007-2012 data are: the total numbers of adult females, males and socially polygynous males, the adult sex-ratio (proportion males), and the numbers of adult females and males deemed available to form new social pairings. Proportions of total adult females and males are shown in parentheses. The number and proportion of males that were socially polygynous decreased from 2007-2009 to 2010-2012, reflecting the changing female-biased to male-biased adult sex-ratio. Also shown are the number of new social pairings that formed in each year and the mean (±1 standard deviation, SD), inter-quartile range (IQR) and range of the coefficient of kinship (*k*_SOC_) across these pairings. The number of new social pairings that formed in a year exceeded the number of females available for pairing when some females formed two new pairings within that year.

| Year: | 2007 | 2008 | 2009 | 2010 | 2011 | 2012 |
| --- | --- | --- | --- | --- | --- | --- |
| Total adult females | 20 | 33 | 21 | 31 | 17 | 37 |
| Total adult males | 13 | 31 | 29 | 36 | 32 | 56 |
| Total (proportion) socially polygynous males | 6 (0.46) | 6 (0.19) | 2 (0.07) | 0 (0) | 0 (0) | 1 (0.02) |
| Total adult sex-ratio | 0.39 | 0.48 | 0.58 | 0.54 | 0.65 | 0.60 |
| Total (proportion) females available for social pairing | 14 (0.70) | 26 (0.79) | 19 (0.90) | 25 (0.81) | 12 (0.71) | 29 (0.78) |
| Total (proportion) males available for social pairing | 11 (0.85) | 29 (0.94) | 24 (0.83) | 30 (0.83) | 26 (0.81) | 46 (0.82) |
| Number of new pairings | 14 | 28 | 22 | 25 | 14 | 32 |
| Mean *k*_SOC_ ± SD | 0.079±  0.027 | 0.110±  0.066 | 0.087±  0.066 | 0.104±  0.055 | 0.120±  0.075 | 0.102±  0.072 |
| IQR of *k*_SOC_ | 0.062-  0.104 | 0.066-  0.137 | 0.053-  0.098 | 0.066-  0.145 | 0.070-  0.125 | 0.065-  0.115 |
| Range of *k*_SOC_ | 0.030-  0.117 | 0.000-  0.301 | 0.000-  0.261 | 0.000-  0.232 | 0.026-  0.308 | 0.000-  0.356 |

**Table S2. Distributions of coefficients of kinship.**

Distributions of individual female song sparrows’ coefficients of kinship (*k*) with the set of males deemed available for social pairing (‘*new-males*’) and all adult males alive in the population (‘*all-males*’) in each focal year spanning 2007-2012, across 125 female-years when new social pairings formed. The numbers and proportions of female-years with zero first-order relatives, second-order relatives or unrelated males available for pairing are given in parentheses. SD and IQR are the standard deviation and inter-quartile range.

|  |  | Mean±SD | Median | IQR | Range | Skew |
| --- | --- | --- | --- | --- | --- | --- |
| Mean *k*_SOC_ | New-males | 0.093± 0.021 | 0.098 | 0.081– 0.110 | 0.040– 0.127 | -0.76 |
|  | All-males | 0.095± 0.021 | 0.102 | 0.084–0.110 | 0.041– 0.130 | -0.83 |
| SD in *k*_SOC_ | New-males | 0.057± 0.016 | 0.059 | 0.047–0.067 | 0.017– 0.094 | -0.44 |
|  | All-males | 0.058± 0.014 | 0.061 | 0.052– 0.067 | 0.019– 0.094 | -0.73 |
| Maximum *k*_SOC_ | New-males | 0.262± 0.068 | 0.284 | 0.214– 0.310 | 0.073– 0.365 | -0.78 |
|  | All-males | 0.277± 0.062 | 0.293 | 0.254– 0.320 | 0.096– 0.368 | -1.14 |
| Minimum *k*_SOC_ | New-males | 0.004± 0.011 | 0.000 | 0.000– 0.000 | 0.000– 0.061 | 3.04 |
|  | All-males | 0.004± 0.011 | 0.000 | 0.000– 0.000 | 0.000– 0.061 | 3.04 |
| Skew in *k*_SOC_ | New-males | 1.04±  0.84 | 1.16 | 0.42 –  1.59 | -1.93–  2.73 | -0.56 |
|  | All-males | 1.25±  0.75 | 1.24 | 0.83 –  1.60 | -0.32–  3.22 | 0.15 |
| First-order relatives | New-males | 1.0±1.0  (45, 0.36) | 1 | 0 – 2 | 0 – 4 | 0.86 |
|  | All-males | 1.3±1.1  (29, 0.23) | 1 | 1 – 2 | 0 – 5 | 0.97 |
| Second-order relatives | New-males | 4.9±3.9  (10, 0.08) | 5 | 2 – 7 | 0 – 16 | 0.87 |
|  | All-males | 5.8±4.7  (8, 0.06) | 5 | 2 – 9 | 0 – 21 | 1.05 |
| Unrelated males | New-males | 2.1±1.0  (14, 0.11) | 2 | 2 – 3 | 0 – 3 | -0.92 |
|  | All-males | 2.2±0.9  (14, 0.11) | 2 | 2 – 3 | 0 – 3 | -1.28 |

**Fig. S1. Distributions of coefficients of kinship relative to the ‘*all-male*s’ set.**

Distributions of the a) mean, b) standard deviation, c) maximum and d) skew in individual female song sparrows’ coefficients of kinship with the ‘*all-males*’ set of all adult males, and the numbers of available e) first-order and f) second-order relatives, across 125 female-years when new social pairings formed.


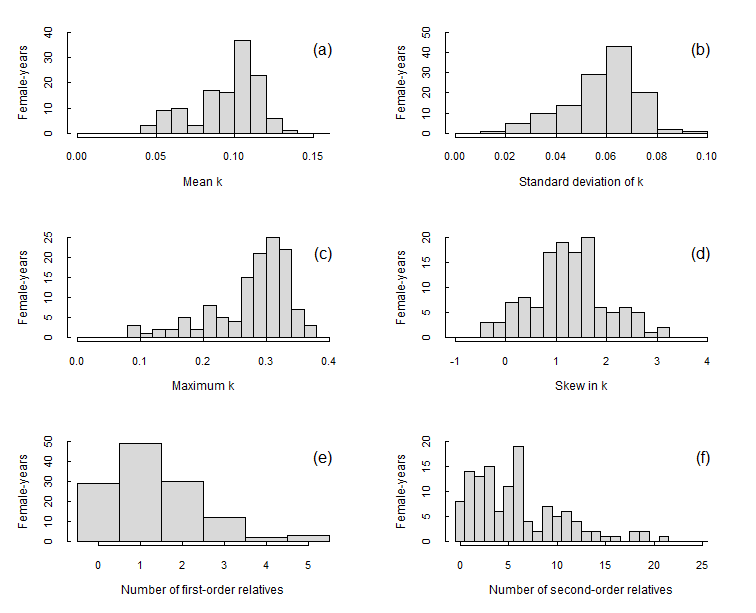


**Fig. S2. Distribution of the deviation in kinship relative to the ‘*all-male*s’ set.**

Distribution of the deviation (*k*_DEV.ALL_) between a female song sparrow’s coefficient of kinship with her observed socially-paired male (*k*_SOC_) and her mean kinship with the ‘*all-males*’ set of all adult males alive in the focal year (*k*_MEAN.ALL_) across 135 observed new social pairings. Mean *k*_DEV.ALL_ was 0.005 ± 0.062 (median -0.004, inter-quartile range -0.023 – 0.019, range -0.114 – 0.235, skew 1.34).


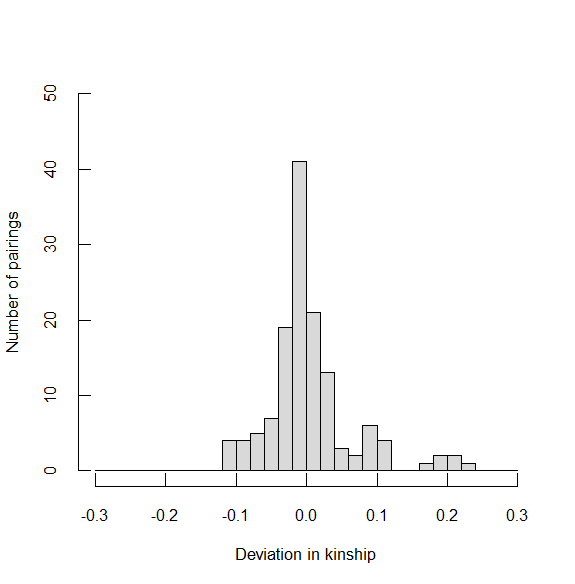


**Fig. S3. Comparison of observed and simulated kinship relative to the ‘*all-male*s’ set.**

Comparisons between the distributions of a) mean coefficient of kinship (*k*_SOC_) between female song sparrows and their observed socially-paired males (dotted line) across 135 newly formed social pairings versus a randomly assigned male from the ‘*all-males*’ set of males alive in each focal year (grey bars), and b) the frequencies of observed (black circles) and simulated pairings falling into categories of *k*_SOC_, defined as 0.025, 0.025-0.05, 0.05-0.075, 0.075-0.10, 0.10-0.125 and 0.125-0.25. x-axis labels demarcate lower category boundaries. Totals of 6, 16, 26, 31, 27, 23 and 6 pairings were observed within these categories respectively. Black bars, boxes, whiskers and circles show the median, inter-quartile range, 1.5xIQR and outliers respectively.


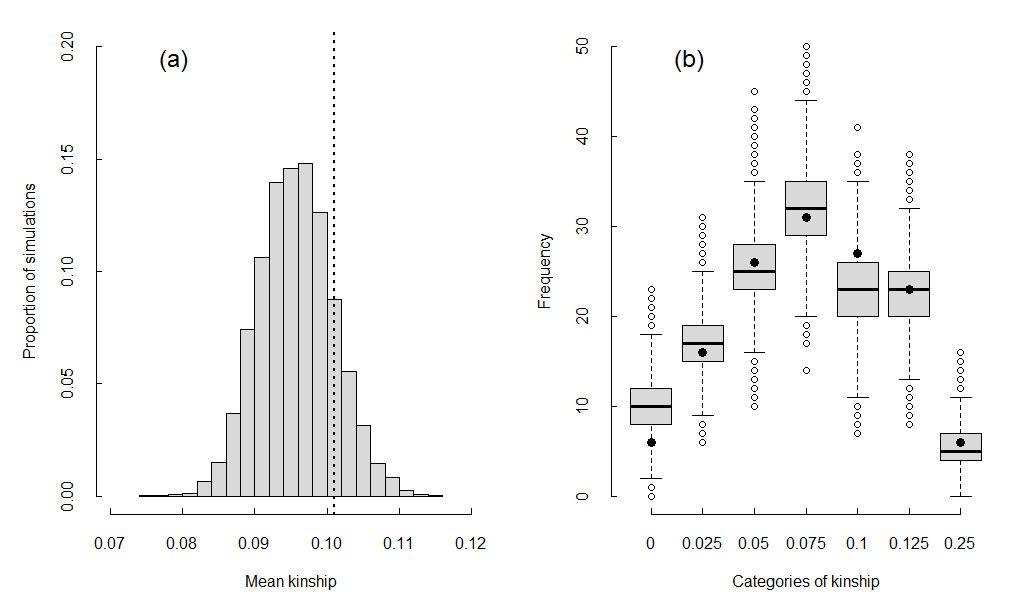


**Table S3. Distributions of coefficients of kinship across years through which social pairings persisted.**

Statistics describing the distributions of individual female song sparrows’ coefficients of kinship (*k*) with all adult males alive in the population (excluding their socially paired male) in each year through which a social pairing persisted, for a) 25 females whose social pairing persisted to a second year and b) 5 females whose social pairing persisted to a third year. SD and IQR are the standard deviation and inter-quartile range.

|  |  |  | Mean±SD | Median | IQR | Range |
| --- | --- | --- | --- | --- | --- | --- |
| a) | Mean | Year of pairing | 0.091±0.023 | 0.102 | 0.076–0.109 | 0.047–0.118 |
|  |  | Second year | 0.093±0.024 | 0.100 | 0.080–0.111 | 0.043–0.130 |
|  | Median | Year of pairing | 0.082±0.025 | 0.095 | 0.068–0.101 | 0.036–0.111 |
|  |  | Second year | 0.083±0.025 | 0.089 | 0.066–0.103 | 0.038–0.119 |
|  | First quartile | Year of pairing | 0.065±0.018 | 0.069 | 0.055–0.080 | 0.031–0.087 |
|  |  | Second year | 0.067±0.020 | 0.071 | 0.056–0.085 | 0.030–0.098 |
|  | Third quartile | Year of pairing | 0.104±0.031 | 0.114 | 0.078–0.126 | 0.047–0.152 |
|  |  | Second year | 0.109±0.032 | 0.114 | 0.088–0.131 | 0.053–0.158 |
| b) | Mean | Year of pairing | 0.103±0.014 | 0.108 | 0.104–0.108 | 0.080–0.116 |
|  |  | Second year | 0.102±0.011 | 0.101 | 0.100–0.110 | 0.086–0.113 |
|  |  | Third year | 0.110±0.009 | 0.109 | 0.107–0.110 | 0.099–0.124 |
|  | Median | Year of pairing | 0.096±0.009 | 0.099 | 0.096–0.100 | 0.081–0.105 |
|  |  | Second year | 0.097±0.010 | 0.096 | 0.096–0.104 | 0.081–0.109 |
|  |  | Third year | 0.100±0.010 | 0.098 | 0.092–0.109 | 0.090–0.113 |
|  | First quartile | Year of pairing | 0.073±0.008 | 0.069 | 0.068–0.082 | 0.066–0.082 |
|  |  | Second year | 0.078±0.005 | 0.078 | 0.073–0.079 | 0.072–0.086 |
|  |  | Third year | 0.072±0.011 | 0.067 | 0.066–0.076 | 0.061–0.089 |
|  | Third quartile | Year of pairing | 0.117±0.015 | 0.119 | 0.114–0.125 | 0.093–0.132 |
|  |  | Second year | 0.121±0.019 | 0.114 | 0.111–0.137 | 0.098–0.143 |
|  |  | Third year | 0.129±0.013 | 0.125 | 0.122–0.139 | 0.114–0.147 |
